# COVID-19 vaccine uptake and hesitation among men and women preparing for pregnancy: a cross-section survey based on the theory of planned behavior

**DOI:** 10.1186/s12889-023-15171-3

**Published:** 2023-02-02

**Authors:** Anjiang Lei, Chunyang Xi, Xiaoxue Luo, Yan Pu, Huaxuan You

**Affiliations:** 1 Department of Obstetrics and Gynecology Nursing, West China Second University Hospital/West China School of Nursing, Sichuan University, Chengdu, Sichuan China; 2 Key Laboratory of Birth Defects and Related Diseases of Women and Children (Sichuan University), Ministry of Education, Chengdu, Sichuan China

**Keywords:** COVID-19, Vaccine uptake, Vaccine hesitation, Preparation for pregnancy

## Abstract

**Background:**

Given the accelerated speed of COVID-19 vaccine research and administration, the main barriers to herd immunity appear to be concerns about safety and efficacy. Men and women preparing for pregnancy may have the same concerns about COVID-19 vaccination, but few studies have focused on COVID-19 vaccine uptake and hesitation among them.

**Methods:**

A cross-sectional study was conducted among men and women who were preparing for pregnancy in Southwest China. The questionnaire was designed based on the theory of planned behavior (TPB). Multiple logistic regression was used to explore the determinants of the behaviors of COVID-19 vaccination.

**Results:**

A total of 2878 participants completed the survey. A total of 53.89% of participants received at least one dose of the COVID-19 vaccine. A total of 45.21% of participants would receive the COVID-19 vaccine in the future. A total of 0.90% of participants never thought about receiving the COVID-19 vaccine. Multiple logistic regression model 1 showed that female participants (OR:5.497, 95%CI: 4.292–7.041), participants who never received influenza vaccine (OR:2.664, 95%CI: 1.908–3.718), participants who had never been tested for COVID-19 (OR:2.244, 95%CI:1.504–3.349), participants who had higher score of negative attitude (OR:1.448, 95%CI: 1.219–1.719), participants who had lower scores of injunctive norms (OR:0.440, 95%CI: 0.360–0.537) and descriptive norms (OR:0.105, 95%CI: 0.088–0.126) were more likely to delay COVID-19 vaccination. Model 2 showed that participants who had lower scores for positive attitude (OR: 0.406, 95% CI: 0.230–0.716), injunctive norms (OR: 0.283, 95% CI: 0.130–0.614) and descriptive norms (OR: 0.060, 95% CI: 0.038–0.094) were more likely to refuse COVID-19 vaccination.

**Conclusions:**

The COVID-19 vaccination rate of men and women preparing for pregnancy was significantly lower than the average vaccination rate of China. Gender, protective health behaviors, vaccination attitudes, and subjective norms had effects on the vaccination behaviors of couples preparing for pregnancy.

## Background

The coronavirus disease 19 (COVID-19) has contributed substantially to excess deaths worldwide since the WHO declared a pandemic [1]. The COVID-19 pandemic has caused a significant threat to the public health system [2, 3]. According to the WHO COVID-19 dashboard, COVID-19 caused over 5.9 million deaths by February 2022 [4]. Developing wide-scale immunity among the population through vaccination is considered the most effective health approach to prevent and control the COVID-19 pandemic [5, 6]. By December 2022, the global fully vaccinated rate is 64.1%, with significant variation across countries and regions [4, 7]. Despite the high efficacy and short-term and medium-term safety of COVID-19 vaccines found in clinical trials, a certain number of people still delay or refuse COVID-19 vaccination [6, 8, 9]. Vaccine hesitation was defined by the WHO as a delay in acceptance or refusal of vaccination despite availability of vaccination services. It was identified as one of the top 10 threats to global health by the WHO in 2019 [10].

Given the accelerated speed of COVID-19 vaccine research and administration, the main barriers to herd immunity appear to be concerns about safety and efficacy [8]. Adverse effects on reproductive health have become a major concern for couples preparing for pregnancy [11]. However, in fact, the International Federation of Fertility Societies advises that women who are preparing for pregnancy have the option to proceed with efforts at conception and to seek a COVID-19 vaccine as soon as possible [12]. The National Health Commission of China also recommends that there is no reason to delay pregnancy attempts because of vaccination administration [13]. These recommendations are consistent with evidence of COVID-19 vaccination on reproductive health. There is evidence that COVID-19 vaccination does not result in any measurable effects on in vitro fertilization (IVF) treatment, including the number of oocytes retrieved, good-quality embryo rate, clinical pregnancy rate and pregnancy outcomes [14]. Likewise, COVID-19 vaccination did not affect the sperm quality and fertilization capacity of men and should be considered safe for men’s reproductive health [11]. Despite the evidence indicating that the COVID-19 vaccine was safe for human reproductive health, some couples seeking care at infertility clinics still hesitate or even refuse to receive a COVID-19 vaccine due to a lack of trust in public health authorities and access to rumors about the COVID-19 vaccine [15–17]. Therefore, men and women preparing for pregnancy may have the same concerns about COVID-19 vaccination.

To explain the vaccination behaviors of our participants, the theory of planned behavior (TPB) was selected as the theoretical framework for this study, which is widely used in predicting and explaining human behavior in specific contexts. As reported, the theory of planned behavior (TPB) has the highest predictive power in determining vaccination behaviors [18]. A previous study also used the TPB as a part of the theoretical framework to explore the determinants of COVID-19 vaccine acceptance among adults [19]. According to the TPB, behavior is driven by the intention to carry out the behavior, ultimately determined by attitudes, subjective norms, and perceived behavioral control. Attitudes toward a behavior can be positive and negative. Subjective norms include injunctive norms (describing how individuals should act from the perspective of family and society) and descriptive norms (describing how families, friends and people around actually act). Perceived behavioral control represents the perceived barriers and self-efficacy of performing a behavior, including internal control and external control [2, 18, 20]. We adapted the original framework slightly to match our research purposes.

Due to a lack of trust in public health authorities and access to rumors about the COVID-19 vaccine, men and women preparing for pregnancy may still hesitate or even refuse to receive a COVID-19 vaccine, but few studies have focused on COVID-19 vaccine uptake and hesitation among them. Along with quantifying the prevalence of COVID-19 vaccine uptake and hesitation, it is crucial to find the determinants of their decision-making process that result in delay or refusal of vaccination so that targeted interventions can be designed to increase COVID-19 vaccine uptake [18].

## Methods

### Study design and sample

This was a STROBE checklist-compliant study. A cross-sectional study was conducted among men and women who were preparing for pregnancy between July 2021 and February 2022 in Southwest China. Invitations to participate in this survey were distributed to the eligible population via hospitals, communities and the Internet. Snowball sampling was used to recruit participants. Considering that the COVID-19 vaccination rate was 64.1% [4], the minimum sample size was calculated as 2213 with a 95% CI and 2% margin of error.

### Measurements

The self-designed questionnaire was based on a literature review and expert consultation. The first section of the questionnaire included information on the sociodemographic characteristics of the participants, such as age, gender, marital status, education level, place of residence, and family per capita monthly income. The second section of the questionnaire included medical insurance, history of influenza vaccination, and testing for COVID-19. The third section of the questionnaire was the 5-point Likert scale based on the TPB, including attitudes (positive and negative attitudes), subjective norms (injunctive and descriptive norms), and perceived behavioral control (internal and external control). The Cronbach α coefficients of attitudes, subjective norms, and perceived behavioral control were 0.788, 0.775, and 0.874, respectively. The global Cronbach’s α coefficient was 0.824, and the construct validity index was 0.909, which showed that this questionnaire had good reliability and validity. Responses range from “Very much agree” to “Very much disagree”. The last question investigated COVID-19 vaccination uptake and intentions. It takes about 5 min to fill out our questionnaire.

### Statistical analysis

The qualitative data were described by composition ratio, and the chi-square test was used for bivariate analysis. The mean and standard deviation (M ± SD) were used to describe the quantitative data with normal distribution and approximately normal distribution, and ANOVA was used for bivariate analysis. Predictor variables (*p* < 0.1) were entered into the multiple logistic regression. Multiple logistic regression was used to explore the influencing factors of the behaviors of COVID-19 vaccination. SPSS23.0 (SPSS Inc., Chicago, IL) was used for statistical analysis. In all analyses, a *p* value of < 0.05 indicated statistical significance.

## Results

### Sociodemographic characteristics and health predictors of COVID-19 vaccination

A total of 2878 participants completed the survey. Participants’ demographic characteristics and health predictors of COVID-19 vaccination are shown in Table 1.

**Table 1:** Sociodemographic characteristics and health predictors of COVID-19 vaccination (N = 2878)

	n (%)
**Age**	
≤ 35	2231 (77.52)
> 35	647 (22.48)
**Gender**	
Male	1347 (46.80)
Female	1531 (53.20)
**Marital status**	
Unmarried	17 (0.59)
Married	2861 (99.41)
**Education level**	
High school or below	1129 (39.23)
Bachelor or above	1749 (60.77)
**Place of residence**	
Rural area	1068 (37.11)
Urban area	1810 (62.89)
**Family per capita monthly income (yuan)**	
≤ 5000	1568 (54.48)
> 5000	1310 (45.52)
**Has medical insurance**	
Yes	2670 (92.77)
No	208 (7.23)
**Has ever received influenza vaccine**	
Yes	371 (12.89)
No	2507 (87.11)
**Has ever been tested for COVID-19**	
Yes	2675 (92.95)
No	203 (7.05)

### TPB variables associated with COVID-19 vaccination

A total of 53.89% (1551/2878) of participants received at least one dose of the COVID-19 vaccine. A total of 45.21% (1302/2878) of participants received the COVID-19 vaccine in the future. A total of 0.90% (26/2878) of participants never thought about receiving the COVID-19 vaccine. The means and standard deviations of TPB variables associated with COVID-19 vaccination are shown in Table 2.

**Table 2:** TPB variables associated with COVID-19 vaccination (N = 2878)

Item	M ± SD/n (%)
**Attitudes**	
Positive attitudes	4.24 ± 0.77
Negative attitudes	2.24 ± 0.70
**Subjective norms**	
Injunctive norms	3.08 ± 0.58
Descriptive norms	4.32 ± 0.82
**Perceived behavioral control**	
Internal control	3.84 ± 0.720
External control	3.87 ± 0.830
**Intentions and Behaviors**	
I never thought about receiving the COVID-19 vaccine.	26 (0.90)
I will receive the COVID-19 vaccine in the future.	1301 (45.21)
I have received at least one dose of the COVID-19 vaccine.	1551 (53.89)

### Bivariate analysis of COVID-19 vaccination

Table 3 shows the associations between the independent variables and the COVID-19 vaccination of participants. Gender, influenza vaccination history, testing for COVID-19 and TPB variables were shown to be associated with COVID-19 vaccination. More details are shown in Table 3.

**Table 3:** Bivariate analysis of COVID-19 vaccination (N = 2878)

	Have received	Will receive	Never thought	χ^2^/F (*p*)
**Age**				**4.949 (0.084)**
≤ 35	1179 (52.85)	1033 (46.30)	19 (0.85)	
> 35	372 (57.50)	268 (41.42)	7 (1.08)	
**Gender**				71.962 (< 0.001)*
Male	833 (61.84)	497 (36.90)	17 (1.26)	
Female	718 (46.90)	804 (52.51)	9 (0.59)	
**Marital status**				**2.825 (0.244)**
Unmarried	1476 (54.11)	1229 (45.05)	23 (0.84)	
Married	75 (50.00)	72 (48.00)	3 (2.00)	
**Education level**				**2.872 (0.238)**
High school or below	597 (52.88)	518 (45.88)	14 (1.24)	
Bachelor or above	954 (54.55)	783 (44.77)	12 (0.69)	
**Residence**				**2.304 (0.316)**
Rural area	593 (55.52)	464 (43.45)	11 (1.03)	
Urban area	958 (52.93)	837 (46.24)	15 (0.83)	
**Family per capita monthly income (yuan)**				**0.562 (0.755)**
≤ 5000	855 (54.53)	699 (44.58)	14 (0.89)	
> 5000	696 (53.13)	602 (45.95)	12 (0.92)	
**Has medical insurance**				**1.050 (0.592)**
Yes	1446 (54.16)	1200 (44.94)	24 (0.90)	
No	105 (50.48)	101 (48.56)	2 (0.96)	
**Has ever received influenza vaccine**				85.941 (< 0.001)*
Yes	283 (76.28)	86 (23.18)	2 (0.54)	
No	1268 (50.58)	1215 (48.46)	24 (0.96)	
**Has ever been tested for COVID-19**				19.906 (< 0.001)*
Yes	1472 (55.03)	1180 (44.11)	23 (0.86)	
No	79 (38.92)	121 (59.61)	3 (1.48)	
**Positive attitudes**	4.35 ± 0.77	4.15 ± 0.74	3.19 ± 1.07	51.615 (< 0.001)*
**Negative attitudes**	2.10 ± 0.71	2.40 ± 0.63	2.72 ± 0.88	77.585 (< 0.001)*
**Injunctive norms**	3.26 ± 0.58	2.89 ± 0.52	2.55 ± 0.65	173.426 (< 0.001) *
**Descriptive norms**	4.77 ± 0.53	3.82 ± 0.78	3.08 ± 1.43	758.026 (< 0.001) *
**Internal control**	4.01 ± 0.71	3.65 ± 0.65	3.01 ± 1.06	114.577 (< 0.001) *
**External control**	4.05 ± 0.82	3.67 ± 0.078	2.85 ± 1.03	100.145 (< 0.001) *

### Multiple logistic regression of COVID-19 vaccination

Table 4 shows the multiple logistic regression analysis of COVID-19 vaccination of participants. These variables in regression models explained 43.9% of the variance in COVID-19 vaccination. Participants who had received the COVID-19 vaccine were set as the reference group. Model 1 showed that female participants (OR:5.497, 95%CI: 4.292–7.041), participants who never received influenza vaccine (OR:2.664, 95%CI: 1.908–3.718), participants who had never been tested for COVID-19 (OR:2.244, 95%CI: 1.504–3.349), participants who had higher score of negative attitude (OR:1.448, 95%CI: 1.219–1.719), participants who had lower scores of injunctive norms (OR:0.440, 95%CI: 0.360–0.537) and descriptive norms (OR:0.105, 95%CI: 0.088–0.126) were more likely to have COVID-19 vaccination hesitancy. Model 2 showed that participants who had lower scores for positive attitude (OR: 0.406, 95% CI: 0.230–0.716), injunctive norms (OR: 0.283, 95% CI: 0.130–0.614) and descriptive norms (OR: 0.060, 95% CI: 0.038–0.094) were more likely to refuse COVID-19 vaccination.

**Table 4:** Multiple logistic regression of COVID-19 vaccination

	β	Wald χ²	*p*	OR	OR(95% CI)
**Model 1** **Will receive/Have received**					
Gender (male = 1, female = 2)	1.704	182.203	0.000*	5.497	4.292, 7.041
Has ever received influenza vaccine (yes = 1, no = 2)	0.980	33.167	0.000*	2.664	1.908, 3.718
Has ever been tested for COVID-19 (yes = 1, no = 2)	0.808	15.658	0.000*	2.244	1.504, 3.349
Positive	0.017	0.047	0.828	1.017	0.873, 1.185
Negative	0.370	17.813	0.000*	1.448	1.219, 1.719
Internal	-0.077	0.469	0.493	0.926	0.743, 1.154
External	-0.183	3.580	0.058	0.833	0.689, 1.007
Injunctive norms	-0.821	65.293	0.000*	0.440	0.360, 0.537
Descriptive norms	-2.254	589.563	0.000*	0.105	0.088, 0.126
**Model 2** **Never thought/Have received**					
Gender (men = 1, women = 2)	0.810	3.000	0.083	2.249	0.899, 5.627
Has ever received influenza vaccine (yes = 1, no = 2)	1.271	2.277	0.131	3.564	0.684, 18.564
Has ever been tested for COVID-19 (yes = 1, no = 2)	0.961	1.932	0.165	2.615	0.674, 10.140
Positive	-0.901	9.709	0.002*	0.406	0.230, 0.716
Negative	0.498	2.248	0.134	1.646	0.858, 3.158
Internal	0.030	0.004	0.948	1.030	0.419, 2.536
External	-0.683	2.918	0.088	0.505	0.231, 1.106
Injunctive norms	-1.263	10.186	0.001*	0.283	0.130, 0.614
Descriptive norms	-2.817	145.691	0.000*	0.060	0.038, 0.094

## Discussion

This study focused on the COVID-19 vaccination behaviors of men and women preparing for pregnancy. In this study, the results revealed that 53.89% of the population had received at least one dose of the COVID-19 vaccine, 45.21% of the population would receive the COVID-19 vaccine in the future, and 0.90% of the population never thought about receiving the COVID-19 vaccine. The vaccination rate was slightly lower than the global average vaccination rate (60.0%) and significantly lower than the average vaccination rate of China (90.0%) [4]. These results verified our hypothesis that a certain number of men and women preparing for pregnancy delayed or even refused to receive the COVID-19 vaccine.

Our results showed that the average vaccination rate for men was higher than that for women, which was inconsistent with previous studies conducted in China and a global survey across 19 countries [21, 22]. We inferred that the reason might be that women preparing for pregnancy are more cautious about vaccines and are concerned about adverse effects. These concerns might be caused by misinformation about the COVID-19 vaccine on social media [23, 24]. Therefore, future health education and science popularization programs should pay more attention to women preparing for pregnancy and provide more correct information about the COVID-19 vaccine to prevent misconceptions from taking hold [23].

We found that participants who never received the influenza vaccine or had never been tested for COVID-19 were more likely to delay receiving the COVID-19 vaccine, which was consistent with a previous study [8]. We inferred that the reason might be health literacy, which is considered to be a contributor to people’s health behaviors [25]. As reported, health literacy was found to be positively related to the capacity to adopt preventive measures such as getting vaccines, testing for COVID-19 and wearing face masks [25, 26]. A previous study also reported that increasing people’s health literacy could lead to reduced vaccine hesitancy [26]. Therefore, vaccine promotion strategies should be tailored for couples preparing for pregnancy, especially those with low health literacy.

We found that participants who were more negative toward the COVID-19 vaccine were more likely to delay vaccination, and participants who were less positive toward the COVID-19 vaccine were more likely to refuse the COVID-19 vaccine. These findings were consistent with previous studies, which reported that attitudes toward vaccines were associated with vaccination intentions [2, 8]. This study confirmed the associations between the attitudes and vaccination behaviors of participants.

We found that injunctive norms (describing how individuals should act from the perspective of family and society) and descriptive norms (describing how families, friends and people around actually act) showed strong effects on vaccination behaviors, including vaccine uptake and hesitation. These findings were consistent with a previous study, which verified the associations between subjective norms (injunctive norms and descriptive norms) and vaccination intention [2]. Based on these findings, we inferred that the vaccination attitudes and behaviors of family and society had effects on the vaccination behaviors of couples preparing for pregnancy. Future health education and science popularization programs should focus on these variables.

We found that perceived behavioral control was not a determinant of behaviors related to the COVID-19 vaccine, which was inconsistent with previous studies focusing on vaccination intentions [27, 28]. We inferred that the reason might be the different populations and different dependent variables. This study focused on the behaviors of COVID-19 vaccination, which might be different from vaccination intentions.

The present study has three main limitations. First, snowball sampling was used to recruit participants, which might have been overestimated due to selection bias. Second, the participants were recruited from Southwest China, which might limit the generalizability of the findings to people from other areas in China or from other countries. Third, our participants also recruited couples undergoing IVF cycles and who might have lower COVID-19 vaccination rates for different reasons, but we did not compare them to normal couples. Future search studies should focus on couples who are undergoing IVF.

## Implications

To achieve herd immunity among the population through vaccination, we need to persuade more people to get vaccinated. This study found that a certain number of men and women preparing for pregnancy delayed or even refused to receive the COVID-19 vaccine. These results could help health authorities in China identify priority populations that need special attention in COVID-19 vaccination campaigns. Our study also identified several influencing factors related to vaccine uptake and hesitancy of couples preparing for pregnancy, including gender, protective health behaviors, vaccination attitudes, and subjective norms. These findings could provide evidence for health authorities in China to design suitable interventions for increasing the vaccine uptake rate.

## Conclusion

The COVID-19 vaccination rate of men and women preparing for pregnancy was significantly lower than the average vaccination rate of China. Women preparing for pregnancy were more likely to delay receiving the COVID-19 vaccine than men preparing for pregnancy. Participants who never received the influenza vaccine or had never been tested for COVID-19 were more likely to delay receiving the COVID-19 vaccine. Vaccination attitudes and subjective norms had effects on the vaccination behaviors of couples preparing for pregnancy. To achieve herd immunity, vaccine promotion strategies should be targeted at these populations and be tailored based on these findings.

## Data Availability

The data that support the findings of this study are available from West China Second University Hospital, but restrictions apply to the availability of these data, which were used under license for the current study and are not publicly available. Data are, however, available from the authors upon reasonable request and with permission of West China Second University Hospital.
